# Design and Development of Smart Weight Measurement, Lateral Turning and Transfer Bedding for Unconscious Patients in Pandemics

**DOI:** 10.3390/healthcare10112174

**Published:** 2022-10-30

**Authors:** Imran Shafi, Muhammad Siddique Farooq, Isabel De La Torre Díez, Jose Breñosa, Julio César Martínez Espinosa, Imran Ashraf

**Affiliations:** 1 College of Electrical and Mechanical Engineering, National University of Sciences and Technology (NUST), Islamabad 44000, Pakistan; 2 National Centre for Robotics and Automation, National University of Sciences and Technology (NUST), Islamabad 44000, Pakistan; 3 Department of Signal Theory and Communications and Telematic Engineering, University of Valladolid, Paseo de Belén 15, 47011 Valladolid, Spain; 4 Higher Polytechnic School, Universidad Europea del Atlántico, Isabel Torres 21, 39011 Santander, Spain; 5 Department of Project Management, Universidad Internacional Iberoamericana Arecibo, Arecibo, PR 00613, USA; 6 Universidade Internacional do Cuanza, Cuito, Bié, Angola; 7 Department of Project Management, Universidad Internacional Iberoamericana, Campeche 24560, Mexico; 8 Fundación Universitaria Internacional de Colombia, Bogota 111311, Colombia; 9 Department of Information and Communication Engineering, Yeungnam University, Gyeongsan 38541, Korea

**Keywords:** pressure ulcers, load cells, hoist lift, patient weight measurement, lateral turning

## Abstract

Patient care and convenience remain the concern of medical professionals and caregivers alike. An unconscious patient confined to a bed may develop fluid accumulation and pressure sores due to inactivity and deficiency of oxygen flow. Moreover, weight monitoring is crucial for an effective treatment plan, which is difficult to measure for bedridden patients. This paper presents the design and development of a smart and cost-effective independent system for lateral rotation, movement, weight measurement, and transporting immobile patients. Optimal dimensions and practical design specifications are determined by a survey across various hospitals. Subsequently, the proposed hoist-based weighing and turning mechanism is CAD-modeled and simulated. Later, the structural analysis is carried out to select suitable metallurgy for various sub-assemblies to ensure design reliability. After fabrication, optimization, integration, and testing procedures, the base frame is designed to mount a hydraulic motor for the actuator, a DC power source for self-sustenance, and lockable wheels for portability. The installation of a weighing scale and a hydraulic actuator is ensured to lift the patient for weight measuring up to 600 pounds or lateral turning of 80 degrees both ways. The developed system offers simple operating characteristics, allows for keeping patient weight records, and assists nurses in changing patients’ lateral positions both ways, comfortably massage patients’ backs, and transport them from one bed to another. Additionally, being lightweight offers reduced contact with the patient to increase the healthcare staff’s safety in pandemics; it is also height adjustable and portable, allowing for use with multiple-sized beds and easy transportation across the medical facility. The feedback from paramedics is encouraging regarding reducing labor-intensive nursing tasks, alleviating the discomfort of long-term bed-ridden patients, and allowing medical practitioners to suggest better treatment plans.

## 1. Introduction

The world is moving towards appliances and gadgets and reducing the use of old devices that use outdated technology. Medical advancement helps to facilitate patients’ rehab by assisting them through different phases of recovery. Modern times require technological assistance for patient recovery; therefore, putting forward challenges to engineering design and technological advancements to aid patients is important. Bodyweight fluctuations are important indications for a range of medical disorders, and in some cases, such as congestive heart failure, they are key determinants of intensive care measures [1]. Many doctors and medical professionals require patient weight for various diagnostic procedures and prescription of medication or diet [2]. Bodyweight, as well as weight change, is particularly important for critically sick patients hospitalized in intensive care units (ICUs), since it is a direct measure of fluid balance effectiveness and general health. About 60% of a male body’s weight is composed of body fluids and, for women, it is 52% [3]. It changes by 1% in a healthy individual. However, if weight change is about 5–10%, it has adverse effects and can cause different diseases such as type 2 diabetes [4], dehydration, and cardiovascular [5] and related diseases. Chittawatanarat et al [6] examined patients’ admission to the ICU, they recorded weight, height, age, and gender. On a regular basis, they monitored for any weight changes and concluded that a variation of more than 5% is an indication of fluid buildup. It is necessary to track changes in body weight regularly to detect fluid abnormalities for the treatment of dialysis [7] and for diagnosing intravenous fluid (IV) treatment problems [8], as well as a nutrient assessment [9]. In Wessex, a survey was performed to find flaws in the process of measuring and documenting patient bodyweight (BW), as well as awareness of weight-based medical calculations in the ICU. Errors and a lack of information can lead to inadequate therapy and the use of potentially dangerous amounts of therapeutic interventions such as LPV. The main aim of this survey was to identify and implement areas of best practice across the Wessex region’s ICUs. The survey’s findings were presented by local leaders who advocated for the documenting of actual bodyweight and height as a requirement for ICU admission. Compliance with documentation of measured body weight and height will be audited following the education process [10].

Recording patient weight is standard practice in hospital admissions. Prescribing narrow therapeutic index medicines may result in significant harm as a result of inaccurate dosing. Patient weight is important for:

- Medical prescription;
- Fluid balance;
- Assessment of nutrition.

Weight measuring for a patient who is either disabled due to some accident or due to older age effects is an important part of medical treatment and is used as a protocol before starting the treatment. So, it becomes important and challenging to have a system in which a patient’s weight can be measured on the bed they are lying in to assist them.

Pressure ulcers (PUs), also known as decubitus ulcers or bedsores, are localized regions of the skin and adjacent tissue injuries caused by applied pressure, friction, or shear [11]. These sores are most commonly found on bony prominences such as the hips, heels, spine, and other joints and are caused by a lack of blood circulation to the area affected for an extended period. Although PUs can form due to a variety of variables including age, nutrition, skin moisture, and general health, they are more commonly encountered in individuals with immobility, spinal cord damage, or other severe conditions, as well as the elderly [12,13]. PUs are still a major health issue that affects millions of individuals. A study shows that PUs were reported in 280,000 hospital stays in 1993, while 455,000 ulcers were reported 11 years later [14]. According to the Healthcare Cost and Utilization Project (HCUP), there was a 63% growth in PUs between 1993 to 2003, but only an 11% increase in overall hospitalizations. Bedsores affect almost 80% of individuals who are confined to beds. In hospitals, the ratio ranges from 0.4% to 38%, and in long-term care facilities, the rate ranges from 2.2% to 23% [15]. Annually, around 60,000 people die as a result of pressure sore complications [16,17]. PUs, alongside a failure to rescue and postoperative respiratory failure, were among the most common complications in the 4th annual “Health Grades Patient Safety in American Hospitals Study”, which examined records from roughly 5000 hospitals between 2003 and 2005. Preventing ulcers has been the sole responsibility of nurses for many years. Clinical guidelines on pressure ulcer prevention were released by the United States Agency for healthcare research and quality in 1992 [18].

Despite considerable advancements, 1300 new ulcers are reported per month only in the United Kingdom, costing about £1.4 million every day. The global challenge is to develop a strategy to improve ulcer prevention and management standards. This is owing to a year-over-year increase of 11% in pressure sores and other types of lesions [19]. In this paper, a hoist-based mechanism is proposed which is capable of turning patients from one side to another to prevent pressure ulcers. In addition, it can be used to measure patient weight efficiently with the help of load cells. It is a simple-to-use system that consists of a motorized hoist that is powered by an actuator that lifts the patient and assists them in turning around. It is a portable solution and can be used for multiple patients across the hospital.

The paper is structured as follows. Section 2 discusses the related works concerning patient-weight-measuring approaches. Section 3 introduces the proposed method, along with its mechanical details, capabilities, and weight-measuring mechanism. Results and discussions are presented in Section 4. Section 5 concludes this study.

## 2. Literature Review

Patient care can be compromised by inconsistencies in patients’ weight measurements, as well as the use of faulty or inadequate weighing equipment [20]. This can raise the chances of diagnosing, intervening, treating, or administering medication incorrectly [21]. Strain gauge-based load cell instruments are the most commonly used commercial ways of determining the body weight of bedridden individuals today. These could be in the shape of a hoist scale or a roll-on scale, in which the patient’s bed is wheeled over the weighing scale, which is mounted to a wall. The other types include detachable units or built-in units. In a clinical context, the detachable units, which must be positioned under each leg of the bed whenever needed, are hard to set up and suffer from usability issues. The inclusion of the cot’s weight to the patient’s body weight in such setups necessitates an over-design of the load cells to ensure excellent accuracy at their lower operating ranges, which also leads to raising the measuring device’s complexity and cost [22]. Without using force sensors, certain experimental techniques for bodyweight assessment are confined to image processing work grounded on the surface area of the body and volume of the elliptic tube [23].

When patients are in a medical emergency or are confined to a bed, it is difficult to measure their weight; as a result, weight is only determined subjectively in these cases. Daniel Benalcazar [24] has proposed an Android-based solution for this to measure weight indirectly using techniques of image processing. For weight estimation, two algorithms, least mean square (LMS) and artificial neural network (ANN), are trained. The preliminary LMS fitting results reveal a significant scattering of data points concerning the fitting curve. The lack of accuracy is due to a variety of factors, including population heterogeneity in terms of apparel and hairstyles. Additional data are fed into the ANN model as an input that assigns labels of different weights depending upon characteristics of hairstyle and apparel. Short hairs and tight clothing are assigned value 1, and an increase in value represents long hairs and loose apparel. Determination of weight depends upon label value and area covered. The study’s findings are found to be impressive, but the user has to enter the hair and clothing data into the algorithm on their own. Labati and co-workers [25] propose another contactless solution to measure weight using the image processing technique by extracting features from a set of frame sequences captured by two cameras. By examining the relationships between the extracted features and the weight of the person, a computational intelligence approach is applied to process the characteristics and estimate the appropriate weight. The results reveal that the approach is viable and capable of estimating weight accurately, having a standard deviation of 2.30 kg and a mean error of 0.07 kg on the analyzed set of frame sequences.

Most of the work focuses on measuring the whole weight of the patient, and region-wise weight measurement is not performed. So, it is hard to track which part of the body is gaining weight. Nancy and colleagues [26] address this problem and propose to measure region-wise weights. The human body is divided into three parts: the head, with 16% height and 7% weight distribution; the trunk, with 27% height and 56% weight distribution; and the legs, with 57% height and 37% weight distribution. Three load cells are connected to steel sheets such that the weight of each part could be measured separately. To obtain a more detailed measurement, three sections are divided into further subsections, each having a load cell underneath it; after appropriate amplification, the weights measured by all 13 load cells are digitally displayed.

Immobility has been found in studies to be a major risk factor in the formation of PUs; nevertheless, the occurrence of PUs is not only influenced by the duration of repositioning [27,28]. This is due to the complex interplay of risk variables that escalates a person’s risk of developing PU rather than a single potential risk that can account for its development [29]. The involvement of nurses in caring for patients who are at risk of PU is a crucial factor. Research findings have shown that the failure of nurses to put their newly acquired information into practice is what actually prevents PU from occurring [30]. Even though PU prevention is characterized as fundamental nursing care, nurses were discovered to be inadequate in its implementation. It requires more than one nurse to reposition a patient, which is labor-intensive. This may have an impact on the growth of PU, so it is important to address the issue of staff compliance with established policies [31].

For years, it has been examined that regularly switching lateral positions for bedridden patients can deliver some benefits and minimize the duration of stay for patients in hospitals [32]. Three methods that are used for patient turning are manual, motorized roller, and hoist-type. Nurses or caregivers manually turning patients over from side to side after every few hours is now the most typical approach for preventing PUs. Parts of a patient’s body can recuperate while the contact pressures between their body and the bed are exerted elsewhere with this manual shifting. However, according to Lyder et al. [33], the system is defective because only roughly 66% of patients receive this treatment on a routine basis, owing to nursing labor shortages. Manual turning requires force by the operator, so it is a crude and cumbersome method; moreover, in this technique, there is a danger of harming the patient. In addition, rolling patients has been implicated in lower back pain in medical practitioners [34]; it also cannot be carried out on those who are critically ill. For this purpose, a powered turning bed is required, which facilitates the patient in turning. Ching-Hua and his team [35] have designed a mechanism for aiding patients to rotate laterally that allows patients to shift lateral position to the right or left within an 80-degree angle, for aiding a patient in moving laterally from one bed to another or from one moving carriage to another, such as a wheelchair and bed transforming to a wheelchair to facilitate patients to get off the bed. The mechanism consists of the main bed and auxiliary bed. The main bed is divided into four sections, operated with the help of electric motors; two of them help patients to sit or tilt their legs. The rest of the two contribute to lifting and horizontal movement when used in combination; the patient can rotate laterally and shift from the main bed to the auxiliary bed. The auxiliary bed consists of three sections: one for the footrest, the second for the buttock, and the third one for back support, and it is capable of transforming into a wheelchair for easy mobility.

A novel idea of a weight measurement system for measuring a person’s body weight at regular intervals is proposed by Manoj et al. [36], which is incorporated with an air-filled mattress. The methodology employs the air pressure in a mattress to track weight changes. It is designed with two distinct application areas in mind: an air-filled pillow for infants and an air-filled mattress for immobile patients. Both systems use a pressure transducer to determine how much weight is being applied. The design is modular, allowing it to be easily integrated into any hospital bed. Air mattresses, on the other hand, are vulnerable to sharp items that are regularly employed in hospital medical care. As a result, mechanical devices [37,38] are preferred that can be integrated or isolated from the rest of the bed by using the bedsheet as a medium to rotate the patient laterally. Mechanical devices may be stiffer and more durable than an air mattress, but an air mattress may be lighter and more flexible.

We also find some interesting commercially available solutions. The Charder MS6000 digital bed scale [39] makes weighing bedridden patients simple and accurate, and it is an important part of dialysis and intensive care. The bed is moved onto two movable beam scales that are put close to the wheels of the bed. The bed’s pre-existing weight is tared, allowing the patient’s actual weight to be accurately determined. It can measure patient weight efficiently but is only confined to weight measuring and cannot be used for rotating patients latterly to prevent pressure sores. Seca’s Electronic Bed Scale 985 [40] is a movable bed and dialysis scale that is great at keeping track of patients’ weight changes. Early detection of weight variations is significant in intensive care units because any shift could jeopardize the patient’s physical well-being. This is an in-bed weighing mechanism that must be zeroed before being used on a new bed. This system is not equipped with a turning feature. The VENDLET V5S [41] is a patient transfer tool that helps patients turn and reposition in bed. The assistive gadget allows for a delicate and seamless relocation of the patient with minimal strain and effort on the part of the healthcare provider. It is a roller-based turning device, expensive, and requires a separate unit for every patient. There is no weighing system integration.

The literature indicates that the proposed solutions are local in nature, costly, and offer a single facility, i.e., either weight measurement or patient lateral turning mechanism. VENDLET V5S uses a motorized roller technique and is capable of turning patients laterally and also transferring the patient from one bed to another efficiently, but it is far too expensive, lacks a weight measurement system, and requires a separate unit for each patient. It is unsuitable for hospitals with large workloads. The cheapest alternative appears to be an air-filled pillow/mattress, but the method has significant practical issues. Air leakage is a big problem, and it is more severe in larger mattresses than in air pillows. The pace at which air leaks is also affected by the outside temperature. Changes in the system’s initial pressure make repeatability testing and calibration challenging. This study endeavors to make a patient turning and weighing bed to overcome the above-mentioned limitations.

## 3. Materials and Methods

**Figure 1:**
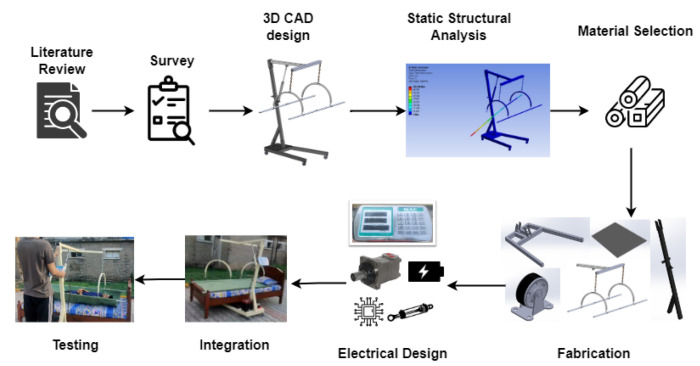
Graphical representation of proposed methodology.

The aim is to develop a low-cost and smart system that could be utilized for various patients around the hospital to prevent bedsores and pressure ulcers, as well as to measure weight. Following a comprehensive review of different types of systems and mechanisms and addressing criteria such as reliability, safety, and simplicity, a hoist-based weighing and turning mechanism is designed and developed. A graphical representation of the proposed methodology is shown in Figure 1.

The first step is to figure out what causes bedsores and errors in weight measurement. Following that, a survey is carried out in several hospitals to determine the average dimensions of patient beds to design a system that would work with beds of various sizes. The CAD model is designed in SOLIDWORKS. The design is finalized after static structural analysis that is conducted on ANSYS to determine design reliability and to check which material best meets our requirements. It is followed by the fabrication and integration of the bed. Table 1 shows the specifications of the proposed system.

### 3.1. Mechanical Design

**Figure 2:**
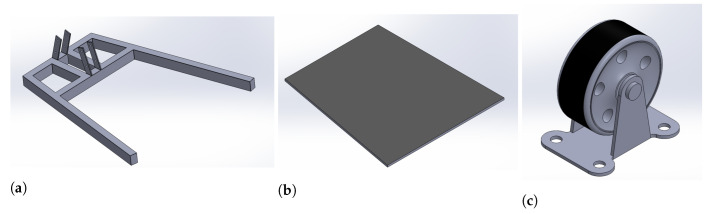
Items used for mechanical design; (**a**) base frame, (**b**) base plate, and (**c**) wheel.

The system is designed to be height-adjustable so that it could be used with beds of different heights. In addition, it should be lightweight and portable, allowing for easy transportation across the hospital. It should be capable of lifting and performing lateral turning of patients up to 270 Kg (600 pounds). The base frame is designed to mount a hydraulic motor, DC battery, and lockable wheels to move the system from one place to another as needed. It is designed with double joints to connect with the upper frame. Figure 2 shows the items used for the mechanical design of the bed.

**Figure 3:**
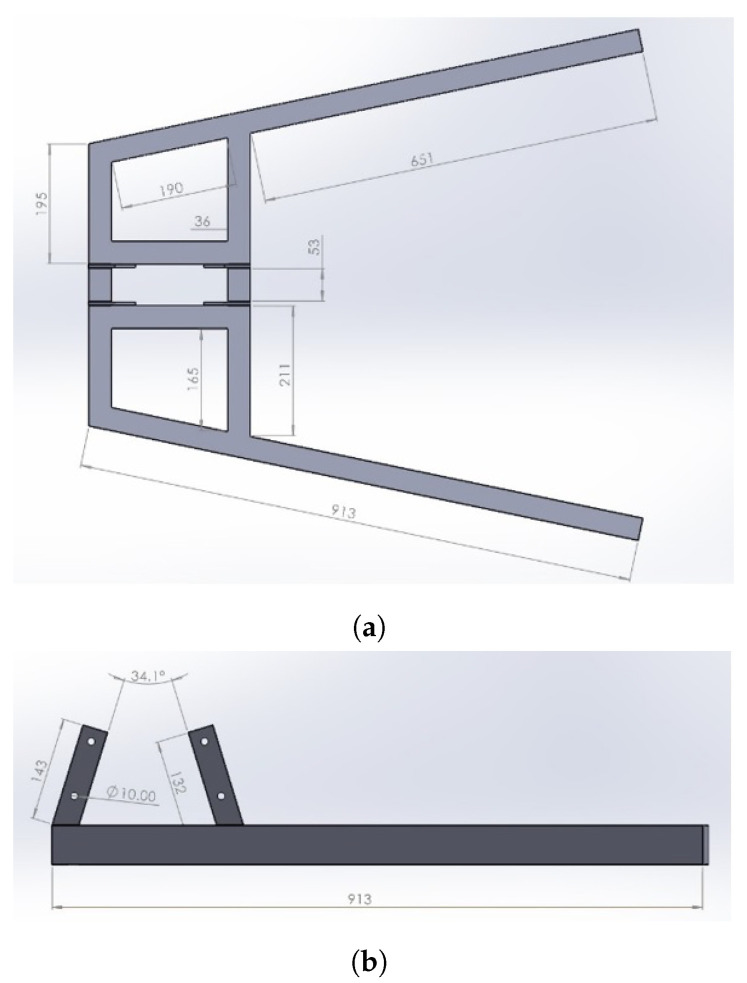
Base frame dimensions; (**a**) top view and (**b**) right view.

Dimensions for the base frame are given in Figure 3.

**Figure 4:**
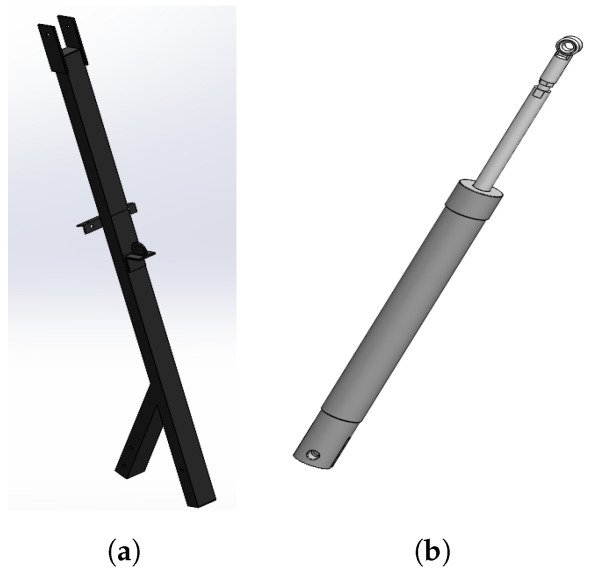
Mount apparatus; (**a**) base main rod and (**b**) hydraulic actuator.

The base main rod is a link between the base frame and the upper support rod. It has a double joint and mounting to incorporate a weighing scale and hydraulic actuator. A hydraulic actuator is used to lift the patient for weight measuring or lateral turning as needed, as shown in Figure 4.

**Figure 5:**
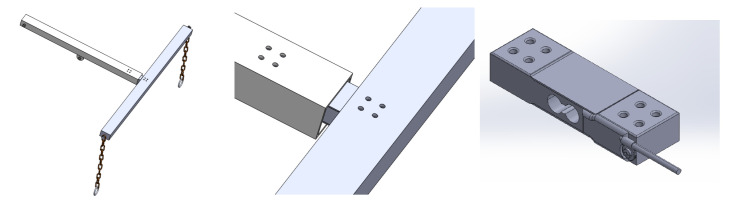
Upper support bars with the load cell.

The upper frame consists of an upper support rod with load cells mounted on it and chains incorporated on both sides for U bar assembly. A strain load cell is used to measure the weight. It is used in *z* formation as a cantilever beam, mounted inside the front upper support rod and upper support rod. The components used for upper support and load cell used for weighing the patient are shown in Figure 5.

**Figure 6:**
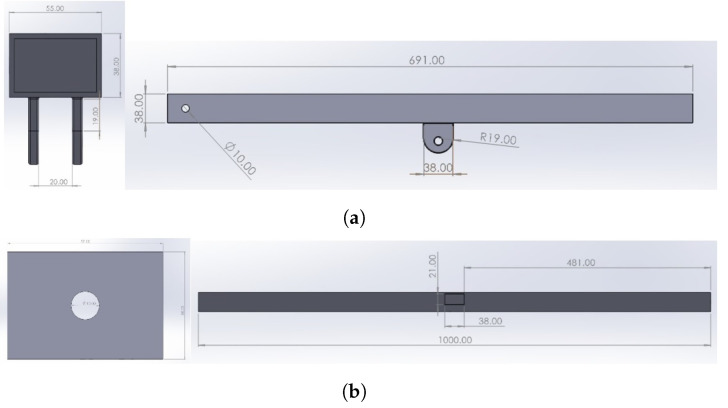
Detailed dimensions for (**a**) upper support bar and (**b**) front support bar.

Dimensions for upper support bar and front support bar are illustrated in Figure 6.

**Figure 7:**
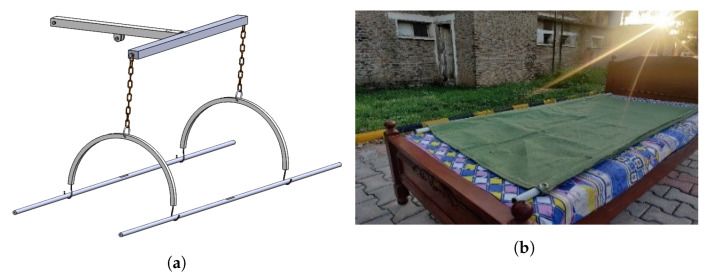
(**a**) Upper frame and (**b**) stretch assembly.

Stretcher assembly consists of two rods and fabric that are laid on the bed and are attached with U bars connected with the upper frame with the help of chains for lifting the patient, as shown in Figure 7.

**Figure 8:**
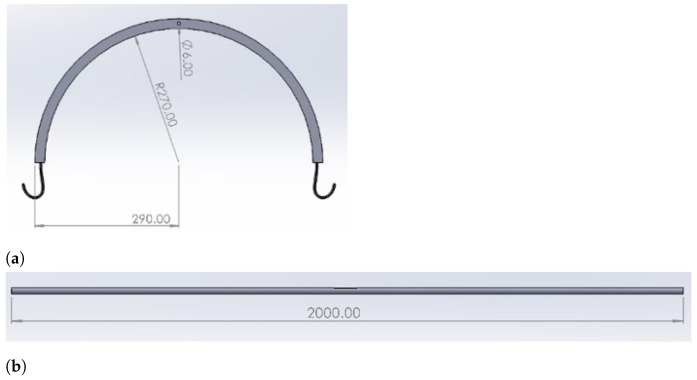
Dimensions of (**a**) U bar and (**b**) stretcher rod.

Figure 8 shows the dimensions for U bar and stretcher rods.

**Figure 9:**
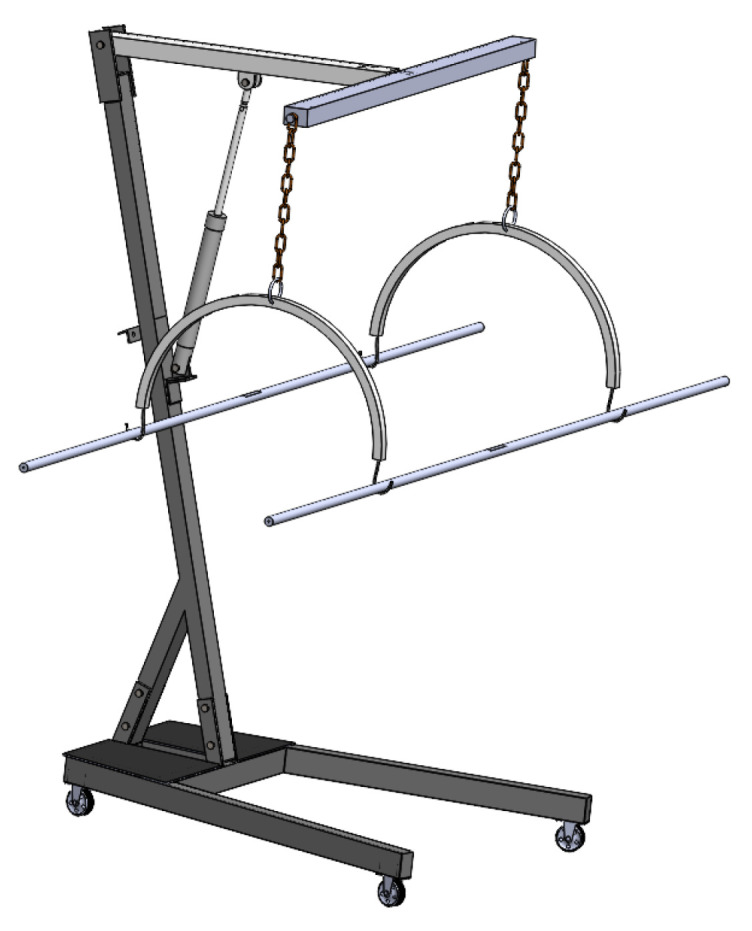
The proposed CAD assembly.

Multiple parts are assembled in Solid works to develop the final CAD assembly. The finalized assembly of the proposed bed is shown in Figure 9.

### 3.2. Numerical Model

**Figure 10:**
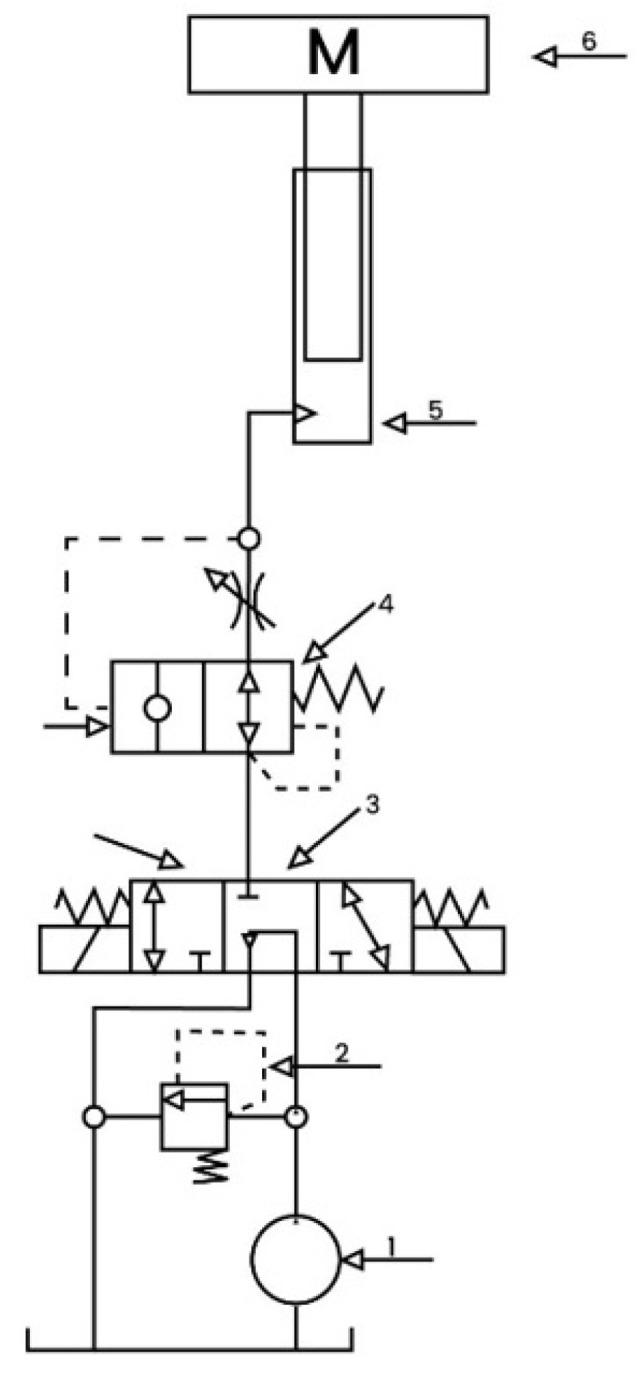
Schematic diagram of hydraulic lift.

A mathematical description of a physical (or other) behavior based on pertinent hypotheses and simplified assumptions is known as numerical modeling. The proposed concept requires an acceleration force to lift the patient, which is generated using a hydraulic actuator. The hydraulic lifting model, as shown in Figure 10, is composed of:

i. Hydraulic pump (power element);
ii. Relief valve (pressure control element);
iii. Directional valve (direction control element);
iv. Explosion-proof valve (speed control element);
v. Plunger-type cylinder (executive element);
vi. Mass (stretcher with the patient).

The hydraulic oil from the hydraulic pump enters through the directional control valve and returns to the oil reservoir in the first motion condition, which is when directional valve 3 is at the mean position. As there is less energy use, the system is unloading. Plunger-type cylinder’s hydraulic feed and return lines are disconnected. Stretcher assembly remains still. When the directional valve goes to the right, the stretcher assembly and plunger-type cylinder move downwards. While the hydraulic pump is not functioning, the hydraulic oil runs in via a plunger-type cylinder, an explosion-proof valve, and a directional valve and returns to the oil reservoir. The hydraulic fluid flowing through the directional valve and explosion-proof valve causes the stretcher assembly to be lifted upward when the directional valve moves to the left.

The model of the proposed system is extended from [42]. The flow rate of the hydraulic pump is given by

(1) $$Q_{p}=vd-C_{p}P_{p}$$

where $Q_{p}$ is the flow rate of the hydraulic pump, *v* is the speed of the hydraulic pump, *d* is the displacement of the hydraulic pump, $C_{p}$ is the internal leakage coefficient of the hydraulic pump, and $P_{p}$ is the outlet pressure of the hydraulic pump.

The flow rate through the relief valve is given by

(2) $$Q_{r}=K_{c}P_{p}$$

where $Q_{r}$ shows the flow rate through the relief valve and $K_{c}$ represents the flow pressure coefficient of the relief valve.

The flow rate through a plunger-type cylinder is given by

(3) $$Q_{i}=A\dot{d}$$

where $Q_{i}$ shows the flow rate through the plunger-type cylinder, *A* is the area of the plunger-type cylinder, and $\dot{d}$ shows the displacement of the plunger-type cylinder.

The outlet pressure of the hydraulic pump can be calculated as

(4) $$P_{p}=\frac{E}{V_{T}}\int \left(Q_{P}-Q_{r}-Q_{i}\right)dt$$

where *E* shows the bulk modulus of the fluid, and $V_{T}$ is the volume occupied between the plunger-type cylinder and pump.

The accelerational force required to lift the patient is given as

(5) $$m\ddot{d}=P_{p}A-mg-f$$

where *m* is the mass of the load (stretcher assembly and patient), *g* shows the acceleration due to gravity, and *f* shows the friction of the plunger-type cylinder.

The friction inside the cylinder is comprised of viscous friction and Coulomb friction. A linear model is used to showcase the friction effects on the force. Thus, the friction model can be described as:

(6) $$f=\left\{\begin{array}{cc} f_{c}sig\left(g\right)+f_{v}\dot{d} & \dot{d}\neq 0\\ f_{s} & \dot{d}=0 \end{array}\right.$$

where *f* shows column friction, $f_{c}$ is the static friction, and $f_{v}$ is the viscous friction coefficient.

### 3.3. Electrical Design

The system is powered by a hydraulic motor that converts electrical power into hydraulic power and is connected to a hydraulic actuator that uses hydraulic power to perform linear actuation and lift the patient. It is operated on 12V DC voltage provided by a rechargeable DC battery. For easy actuation, the hydraulic motor is triggered by a push-button mounted on the main base rod. To measure weight, a weighing scale is used and is connected to a load cell. A voltage is applied to the load cell, and the weight causes the current through it to change. The current is converted to a digital number by an analog-to-digital converter, translated by digital logic to the correct units and displayed on the display. It is mounted on the upper frame, connected with a load cell. Calibration of the scale is performed at up to 270 kg (600 pounds) and, upon startup, it gives self-zero. The specifications of the hydraulic motor are given in Table 2.

**Figure 11:**
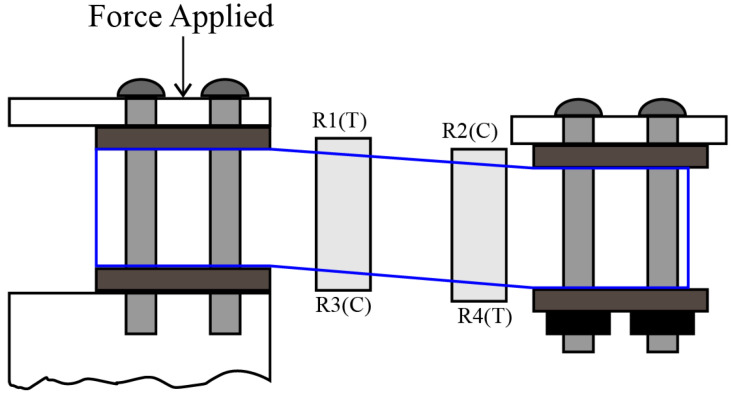
Force applied on *z* formation bar load cell.

To measure weight bar strain, a load cell is used. The load cell is set up in *z* configuration, so that when a force is applied it creates torque, and the strain gauges that are integrated with the load cell measure bending distortion by measuring tension and compression. Out of four two-measure tensions, two of them measure compression, as shown in Figure 11. They convert a force such as tension, compression, pressure, or torque into an electrical signal that can be measured and standardized. As the force applied to the load cell increases, the electrical signal changes proportionally. It is mounted inside the upper frame and connected with a weighing scale to display the measured weight. Calibration is performed with 1 kg of weight, and the maximum rating is 200 kg.

### 3.4. Material Selection

**Figure 12:**
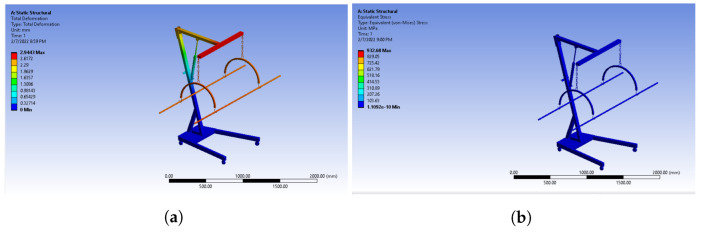
(**a**) Equivalent stress and (**b**) total deformation using stainless steel.

**Figure 13:**
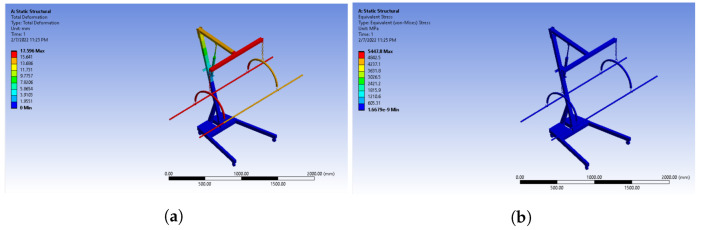
(**a**) Equivalent stress and (**b**) total deformation using aluminum.

Material selection is an important step, as the durability of the system greatly depends on the material. Keeping all the constraints in mind, two materials are focused on for this system, including aluminum and stainless steel. To determine the optimal material for our system, we used ANSYS to run a static structural analysis. The analysis is performed on all materials using finite element analysis (FEA). FEA is a numerical approach for calculating the system’s maximum stress and strain under specified boundary and loading conditions. Because the wheels have to be locked while elevating the patient for weight measurement, they are given fixed support in ANSYS. The downward force is applied to the stretcher rods to represent the weight of the body. Equivalent stress, elastic strain analysis, and total deformation are shown in Figure 12 and Figure 13, respectively.

The results of the static structural analysis clearly show that stainless steel is suitable for our needs. Therefore, stainless steel is selected for fabrication. High-density polyethylene fabric (HDPE) is used for stretchers as it provides greater structural flexibility than other forms of polyethylene. Chemically, the increased density is due to reduced branching in the fabric’s molecular composition. This comprehension results in greater molecular stresses in tension fabric membranes and a significant strength-to-density ratio. The physical properties of HDPE are shown in Table 3.

### 3.5. Fabrication and Integration

Stainless steel is used for fabrication due to its durability and lightweight. It is impossible to build the entire system in one piece; we had to build it in parts to meet our design criteria. To create an assembly, the parts are then assembled according to the design. To develop a complete system, mechanical and electrical components are integrated. A hydraulic motor is used for the lifting mechanism, which is installed on the base and coupled to a hydraulic actuator. A hydraulic actuator is fitted at a 45${}^{\circ }$ angle between the upper frame and the base main rod. A load cell is integrated with the upper frame between the front upper support rod and the upper support rod for weight measurement. The load cell is coupled to a patient weighing system that transforms electrical signals into weight and displays it on an LCD.

## 4. Results and Discussions

**Figure 14:**
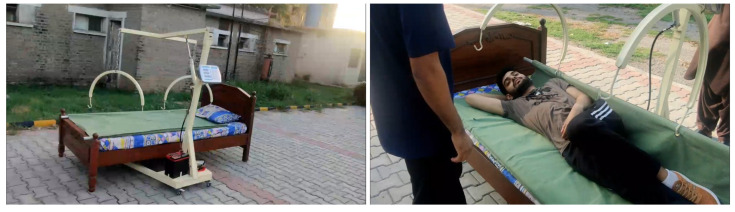
Setting up the device for lateral turning.

It is a simple yet efficient system that consists of a hoist powered by the hydraulic motor via a hydraulic actuator that provides power to the hoist mechanism to lift the patient and help them turn around. The system is simple to operate, and a weighting measuring mechanism is also incorporated into it. Weight is measured by using a lift mechanism where the patient is lifted and the weight is measured with the help of a load cell. It is powered by a single 12 V rechargeable DC battery and features an automated shut-off feature. The hydraulic motor moves the patient up using the U bars, which are in turn supported by the upper frame; the scale also has a membrane keypad with three keys: ON/ZERO, OFF, and LB/KG and features high readouts. It is very easy to set up and operate; the procedure is quickly learned. The lock-in weight feature stores the last reading when the unit is turned off. For lateral turning of a patient, wheels should be locked so that the system should remain stationary. Connect the U bars with a stretcher from one side; the other side should not be connected, as shown in Figure 14. After this, start the hydraulic motor by pressing a button mounted on the main base rod. The hydraulic motor moves the U joints up and down through hydraulic pressure in the upper frame assembly, which then turns the patient. The system is capable of turning a patient from one lateral position to another lateral one and from lateral position to supine position with the resolution of 5-degree rotation.

**Figure 15:**
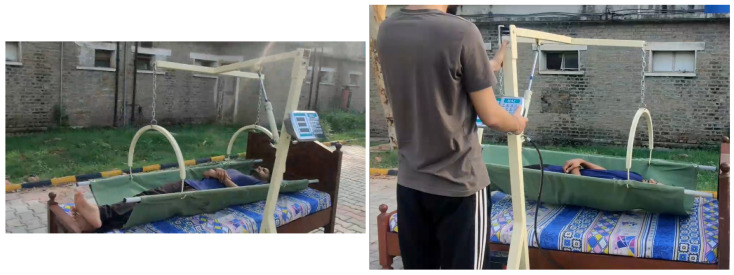
Weight measuring using the designed device.

To measure weight, connect the U bars on both sides of the stretcher placed beneath the patient and press the button to trigger the hydraulic actuator which lifts the patient from the bed, as shown in Figure 15, and force will be applied on the load cell, and the corresponding weight is displayed on the LCD.

It is a legal necessity in care environments to have precise and error-free weighing equipment. Slight variations in weight may be clinically meaningful, hence hospital weighing scales have to be in the Class III category. Testing results of our system show an accuracy of about 0.01 kg, hence it lies in the Class III category. With a patient weighing 270 kg, the testing trial for weight measuring and lateral turning was successful. Just by pressing a button, nursing staff are able to rotate patients laterally, as compared with manual turning which requires a lot of effort. As illustrated in Table 4, the proposed solution is a low-cost portable solution that can be used for multiple patients and is capable of efficiently measuring weight and turning patients latterly. However, on the other hand, commercially available solutions are far too expensive and cannot perform both operations.

## 5. Conclusions and Future Work

Patient care and convenience are the concern of medical professionals, and their importance has been elevated during the COVID-19 pandemic. Unconscious and bedridden patients may develop fluid accumulation and pressure sores due to inactivity and deficiency of oxygen flow. So, their time-to-time turning is critical, as is weight monitoring for effective treatment. Existing commercial solutions are expensive and predominantly do not support both operations. To overcome such limitations, a low-cost and contactless solution for patient weight monitoring and lateral turning is presented. It is simple to use, lightweight, portable, and can be used for multiple patients across the hospital. In pandemic situations such as COVID-19, patient handling requires the highest standards for the safety of healthcare workers. The proposed hardware provides precise weight measurements with a resolution of 0.01 kg and a load capacity of 270 kg (600 pounds). It is evident from experimental results that the patient was successfully repositioned from either lateral position to a supine position or one lateral position to another lateral position, with a minimum resolution of 5 degrees of rotation. The stretcher was also successfully tested to have the necessary strength for maximum weight measuring. The experimental results demonstrate the effectiveness of the approach.

## Figures and Tables

**Table 1:** Specifications of the proposed system.

Feature	Specification
Working Load	270 KG
Power Rating	12V DC
Weight Monitoring	Yes
Lateral Turning	Yes
Portable	Yes
Can be utilized for multiple beds	Yes

**Table 2:** Specifications of hydraulic motor.

Feature	Specification
Operating Voltage	12V DC
Standard Stroke	25–800 mm
Maximum Thrust	6000 N
Speed with no load	5 mm/s
Working Temperature	−26 ${}^{\circ }$ C to 65 ${}^{\circ }$ C
Limit Switch	Built in

**Table 3:** Physical properties of high-density polyethylene fabric.

Parameter	Value
Density	0.954 g/cm ${}^{3}$
Yield strength	20–30 MPa
Ultimate tensile strength	26 MPa
Toughness	2–5 MPa
Elastic modulus	1.0 × 103 MPa
Working temperature	−80 ${}^{\circ }$ C–100 ${}^{\circ }$ C

**Table 4:** Comparison of commercially available solutions.

Device	Weight Limit	Turning	Cost
Vendet VS5	200 kg	Yes	USD 10,980 + GST
Seca 985 Electronic Bed and Dialysis Scales	250 kg	No	USD 6320
Detecto IB800 Digital Stretcher Scale	350 kg	No	USD 10,757
**Proposed Solution**	**270 kg**	**Yes**	**USD 1500**

## Data Availability

Not applicable.
